# Preliminary Study on Wearable Smart Socks with Hydrogel Electrodes for Surface Electromyography-Based Muscle Activity Assessment

**DOI:** 10.3390/s25051618

**Published:** 2025-03-06

**Authors:** Gabriele Rescio, Elisa Sciurti, Lucia Giampetruzzi, Anna Maria Carluccio, Luca Francioso, Alessandro Leone

**Affiliations:** Institute for Microelectronics and Microsystems, National Research Council of Italy, 73100 Lecce, Italy; gabriele.rescio@cnr.it (G.R.); luciagiampetruzzi@gmail.com (L.G.); annamaria.carluccio@imm.cnr.it (A.M.C.); lucanunzio.francioso@cnr.it (L.F.); alessandro.leone@cnr.it (A.L.)

**Keywords:** surface electromyography, wearable device, conductive hydrogel electrodes, health technology, muscle monitoring, smart systems

## Abstract

Surface electromyography (sEMG) is increasingly important for prevention, diagnosis, and rehabilitation in healthcare. The continuous monitoring of muscle electrical activity enables the detection of abnormal events, but existing sEMG systems often rely on disposable pre-gelled electrodes that can cause skin irritation and require precise placement by trained personnel. Wearable sEMG systems integrating textile electrodes have been proposed to improve usability; however, they often suffer from poor skin–electrode coupling, leading to higher impedance, motion artifacts, and reduced signal quality. To address these limitations, we propose a preliminary model of smart socks, integrating biocompatible hybrid polymer electrodes positioned over the target muscles. Compared with commercial Ag/AgCl electrodes, these hybrid electrodes ensure lower the skin–electrode impedance, enhancing signal acquisition (19.2 ± 3.1 kΩ vs. 27.8 ± 4.5 kΩ for Ag/AgCl electrodes). Moreover, to the best of our knowledge, this is the first wearable system incorporating hydrogel-based electrodes in a sock specifically designed for the analysis of lower limb muscles, which are crucial for evaluating conditions such as sarcopenia, fall risk, and gait anomalies. The system incorporates a lightweight, wireless commercial module for data pre-processing and transmission. sEMG signals from the Gastrocnemius and Tibialis muscles were analyzed, demonstrating a strong correlation (R = 0.87) between signals acquired with the smart socks and those obtained using commercial Ag/AgCl electrodes. Future studies will further validate its long-term performance under real-world conditions and with a larger dataset.

## 1. Introduction

In recent years, surface electromyography (sEMG) has received increased clinical interest due to technological advances that have evolved its attractive application in several different fields. Medical applications such as assessing age-related changes in gait, diagnosing conditions like Sarcopenia Pathology, or neuropathies are considered the heightened focus, among population, such as the maintaining musculoskeletal health [1,2]. From this perspective, sEMG offers a valuable tool for assessing muscle function and designing personalized rehabilitation programs for athletes, sports professionals and also for aging people; sEMG could optimize training techniques, improve athletic performance and prevent injuries or dangerous falls [3]. According to the World Health Organization (WHO), falls are the second leading cause of accidental injury deaths worldwide, with individuals aged 65 and older being particularly vulnerable [4]. This alarming trend together with the necessity of advanced sensory systems capable of capturing information related to the activity of the human nervous system, has led to a growing focus on health monitoring, and on effective, user-friendly and challenging technology as a critical tool in this endeavor [5,6,7]. In this context, muscle synergy analysis has also been explored as a quantitative metric for assessing fall risk, identifying compensatory motor patterns, and guiding the development of assistive devices [8,9].

Surface EMG is a technique used to record and analyze the electrical activity produced by skeletal muscles, as electrophysiological signals that are attractive outcomes with several applications in medicine [10]. Unlike invasive intramuscular EMG, which requires the insertion of fine needle electrodes into the muscle tissue, sEMG involves the placement of surface electrodes on the skin above the target muscle groups. These electrodes detect and measure the electrical signals generated by the muscles during contractions, providing valuable insight into muscle function and activity [11].

Despite the advancements in sEMG applications, several technological challenges must be addressed to ensure reliable and practical use in real-world scenarios. One of the most critical issues is the precise positioning of electrodes, which is essential to obtain high-quality signals from specific muscle groups. Traditional sEMG systems rely on commercial pre-gelled electrodes that must be placed at precise anatomical locations, often requiring the intervention of a trained caregiver or clinician. This dependence makes them less suitable for long-term, at-home monitoring, where users may lack the necessary expertise to position the electrodes correctly. Additionally, even minor deviations in placement can lead to variations in signal amplitude and frequency content, reducing the reliability of data acquisition over time.

Another fundamental issue is high skin–electrode impedance, which directly influences the signal acquisition quality. While commercial Ag/AgCl electrodes benefit from conductive gel to reduce impedance, their prolonged use can cause skin irritation and dehydration, leading to a degradation in signal quality. Dry electrodes and textile-based alternatives have been proposed to improve user comfort, but they often suffer from higher impedance and increased susceptibility to motion artifacts. Conductive hydrogels, such as those employed in this study, offer a promising compromise by providing a low-impedance interface while maintaining flexibility and biocompatibility.

User comfort and long-term wearability also present significant challenges in sEMG applications. Wearable systems must strike a balance between electrode adhesion, durability, and breathability to ensure prolonged use without causing discomfort or skin irritation.

By integrating sEMG devices into wearable systems or clothing, muscle activity patterns during walking can be captured and analyzed for gait parameters such as step length, stride time, and muscle activation timing [2]. These measurements help to identify deviations from normal gait patterns widely indicating potential issues in rehabilitation, prosthetics, sports science, and diagnostic applications. In particular, falls often occur due to a combination of factors, including declining muscle strength, impaired balance and coordination, gait abnormalities, and delayed reflexes. Surface EMG devices offer a non-invasive and convenient means to measure muscle activity patterns associated with these risk factors.

The development of wearable devices for EMG involves the use of electrodes capable of capturing signals under dynamic conditions, along with an electronic module to read and transmit the data.

Various types of electrodes have been developed for sEMG [12], including the Ag/AgCl gel electrodes most commonly used in clinical practice [13], conductive polymer electrodes [14], and also dry electrodes such as solid metal electrodes [15] and textile electrodes [16,17].

Table 1 shows the different wearable systems incorporating electrodes for surface electromyography investigated in the literature in recent years. Most of these systems use textile electrodes embedded within the garment. For example, in [18], the authors compared seven different textile electrodes embedded in a sock with Ag/AgCl electrodes and showed that the signal acquired with the textile electrodes had a large amount of background noise and the skin–electrode resistance was higher than that with the commercial electrodes. In [19,20] the authors use sewn or clipped textile electrodes with a button in a sock, simplifying the electrode placement. However, the downsides are that the detected signal differs in amplitude compared with that from commercial electrodes and is very noisy. Additionally, the system lacks biocompatibility, as it contains nickel components that can cause allergic reactions. In contrast, the author of [21] uses electrodes fabricated from carbonized foam embedded in a thin band worn on the calf. This setup offers advantages in signal cleanliness, as the use of carbonized foam electrodes reduces power line interference compared with Ag/AgCl electrodes. However, the correct positioning of the band, and consequently of the electrodes, proves to be more challenging.

Conductive hydrogels, on the other hand, are proposed as a promising alternative for sEMG due to several key advantages demonstrated in the literature.

These hydrogels are typically fabricated with biocompatible polymers and are more skin-friendly than traditional metal electrodes, reducing the risk of irritation and allergic reactions during prolonged use [22]. This makes them particularly suitable for continuous and long-term monitoring, which is essential for applications in rehabilitation; health monitoring; and the diagnosis of conditions like sarcopenia, postural issues, and sclerosis [23,24,25].

In addition, the soft and flexible nature of the hydrogels allows them to adhere better to the skin’s surface than metal electrodes, providing greater comfort, especially for older patients or those requiring longer monitoring periods, reducing motion artifacts and leading to reduced impedance at the interface [26,27,28].

In this work, we propose a wireless smart sock for sEMG, designed to monitor the contractions of the Gastrocnemius Lateralis (GL) and Tibialis Anterior (TA) muscles. Hybrid polymer electrodes consisting of a conductive textile fabric embedded in a conductive hydrogel were developed. The adhesive properties, inherent flexibility, biocompatibility, and high comfort of hydrogels were combined with the performance of conductive fabrics to facilitate their incorporation into various wearable, woven or seamlessly stitched garments. An elastic sock, which incorporates the developed electrodes and all electronic components, constitutes our wearable sEMG data acquisition and transmission system. The smart socks seem to be a readily available solution in the manufacturing and embedding of electrodes in garments and have been shown to yield promising results for signal quality, noise reduction during the acquisition, and the facilitation of the automatic placement of electrodes at points of interest (the muscle groups being observed). The cost and the manufacturing complexity are significant considerations too. Although, initially, these electrodes could require investment in specialized materials and manufacturing processes, they can be produced more cost-effectively at scale. This makes them a more attractive option for mass production and widespread use.

**Table 1:** Wearable system sEMG detection.

Author, Year, References	Wearable System	Type of Electrodes	Electrodes Placement	Biocompatibility
Nathan R. Lyons et al. (2023) [18]	Calf band	7 sets of commercial conductive texiles electrodes: sourced from lessEMF^®^ (Less EMF Inc., Latham, NY, USA)	Some electrodes were embedded into garment via a stretchable heat transfer vinyl (SportFlex, Cricut (Cricut Inc., South Jordan, UT, USA)). One electrode was ironed directly onto the substrate material.	✓ Less. Nickel-/copper-plated polyester textile electrodes could cause skin allergy.
Eguchi et al. (2017) [19]	Stretchy sock (Dr.Sholl, Medi-Qtto^®^ (Scholl’s Wellness Company LLC, Chicago, IL, USA) short sock)	Fabric electrodes (Nishijin electrodes AGposs^®^ (Mitsufuji Corporation, Kyoto, Japan))	Fixed to socks using double-sided fusible tape. In addition, the male part of a nickel hook was caulked.	✓ Less. Nickel hook could cause allergy on prolonged use.
Eguchi et al. (2019) [20]	Stretchy long sock	Fabric electrodes (Nishijin electrodes AGposs^®^)	Fixed in the sock; the difference from the previous one is the specially sewn knee adaptor parts, in which the forward and back directions are easily distinguishable by sight.	✓ Less. Large male-side part consisting of a rivet snap fastener made of brass.
Khokhlova et al. (2020) [29]	Leggings	Constructed texile electrodes	Conductive fabric (nylon silver-coated woven fabric) glued onto the basic fabric and fixed with a sewing machine.	Conductive fabric suitable for medical application.
Bao et al. (2018) [21]	Multifuncional lower limb band	Carbonized foam electrode (band)	Li-battery power supply module, an inertial measurement unit (IMU, Invensense MPU9250 (TDK InvenSense, San Jose, CA, USA)) module and an EMG module with carbonized foam electrode were embedded in the multifunctional band.	✗ Not indicated. Carbonized foam surface should have high biocompatibility.
Cerone, et al. (2021) [30]	Textile grid of silver electrodes integrated into a sleeve applied to the shank	A wearable HD-sEMG detection system composed of 32 electrodes with 15 mm Inter-Electrode Distance (IED)	A layer of silver electrodes and conductive traces deposited on a stretchable thermoplastic-polyurethane (TPU) support (IntexarTM^®^ Intexar™ (Celanese Corporation, Irving, TX, USA)).	✗ Not indicated. Plastic should affect the transpirability.
Shafti 2016 [31]	A designed wearable system for grip muscle analysis	A stainless steel conductive thread	The stainless steel conductive thread was sewn into the fabric with a regular non-conductive thread, to hold it in place.	✗ Not indicated. However, nickel traces should be not present, and so the biocompatibility should be higher than in other conductive threads.
Isezaki T. 2019 [32]	A sock-type weareble sensor	A knit conductive fabric made of silver-plated nylon (available from SparkFun) electrode	The conductive knit fabric is fixed in the sock with snaps located at the center of the electrodes.	✗ Not indicated. However, the snaps generally made of brass should affect the biocompatibility.
Ankit Vijayvargiya [33] 2022	Not wearable, medical-grade electrodes on skin	Custom electrodes: MYOWARE^®^ 2.0 (Advancer Technologies, Raleigh, NC, USA) Muscle sensor with pre-gelled Ag/AgCl electrodes	A system composed of a ESP32-Wroom-32 (Espressif Systems, Shanghai, China) module linked to the outputs of two MyoWare muscle sensors (two-channel system).	✗ Less. Custom pre-gelled Ag/AgCl electrodes could cause burns and allergic reaction in the skin.
Cerone et al., 2019 [34]	A miniaturized, wireless, and modular HD-sEMG acquisition system	A flexible Kapton© (DuPont, Wilmington, DE, USA) grid of 32 Ag electrodes	Sensor Unit (SU) module is enclosed in a 3D-printed PLA case and connected to a flexible Kapton© grid of 32 Ag electrodes.	✗ Not indicated. However, Kapton is commonly used in medical fields for its biocompatibility and long-time stability.

## 2. Materials and Methods

The proposed system is embedded within a sock and is designed to capture sEMG signals from the leg, specifically targeting the GL and TA muscles, during normal daily activities. The key features of this wearable device include ease of use, lightweight design, and biocompatible electrodes positioning over the target muscles, all of which enable comfortable, continuous, and long-term monitoring.

### 2.1. Hardware Architecture

**Figure 1:**
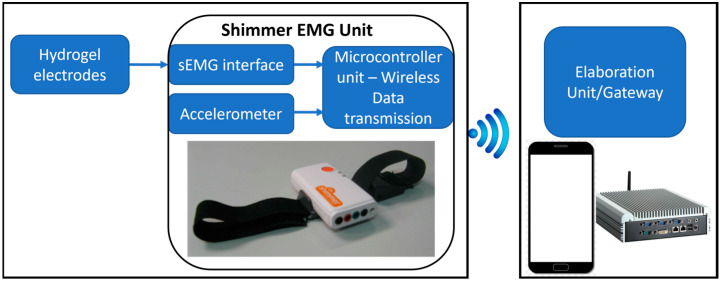
Overview of the hardware architecture.

The developed device primarily consists of (a) compression stockings, (b) hybrid polymer electrodes (HPes) that maintain contact with the skin, and (c) an electronic module for reading and transmitting data from the electrodes. Figure 1 provides an overview of the smart socks.

Soft, conductive composite hydrogels based on sodium alginate and graphite were developed as wearable electrodes for sEMG measurements. Alginate was used for its excellent biocompatibility and gelling ability through ionic cross-linking with calcium ions [35,36]. Conductive graphite was dispersed in the hydrogel matrix, and a flexible conductive fabric was incorporated to support the Ag/AgCl snap button. Hybrid polymer electrodes were chosen to ensure good adhesion to the skin and low skin–electrode impedance.

To optimize electromyographic signal readings, compression stockings that fit the calf well were considered. Various types of compression stockings were evaluated, including graduated or medical compression stockings, anti-embolic stockings, and non-medical support stockings. The non-medical model was selected due to its elasticity and suitability for prolonged use without discomfort. These stockings, commonly used in sports or for relieving tired, heavy legs, provide even and significantly lower compression than medical-grade alternatives, typically around 15–20 mmHg, and are easily available, cost-effective, and do not require a prescription [37]. This makes them ideal for long-term preventive monitoring. The chosen stockings, made from breathable elastic technical yarns (84% polyamide, 16% elastane), offer good electrode–skin coupling and comfort during physical activity and daily wear. Available in multiple sizes, they can be hand or machine washed at 30 °C.

To capture and amplify signals from the hydrogel electrodes, the Shimmer3 EMG unit (shown in Figure 1) was used [38]. This unit features a customizable digital front-end designed for precise electromyographic signal acquisition, detecting muscle contractions and integrating a sensor for monitoring leg movements. This allows for additional analysis of body stability and balance or the identification of EMG artifacts caused by motion. The device offers software-modifiable gain (up to 12), making it compatible with electrodes of varying types and sizes. Compact (65 × 32 × 12 mm) and lightweight (31 g), the device transmits data wirelessly to a processing unit (e.g., notebook, smartphone, tablet) in real time or stores data onboard for offline analysis. A detailed overview of these features and others is provided in Table 2.

Shimmer devices are equipped with a comprehensive software suite for data storage and analysis, as well as with software development kits (SDKs) in multiple programming languages (e.g., Matlab, Python, C), allowing for the creation of fully customized applications, including real-time data processing. The Shimmer3 unit features two channels for EMG signal acquisition and includes a reference electrode, enabling the simultaneous monitoring of two muscles.

**Figure 2:**
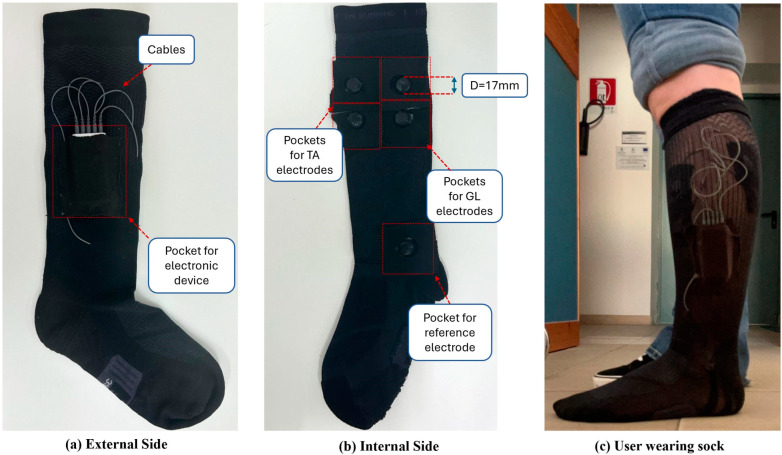
Overview of the socks. (**a**) External side of the socks: equipped with a pocket for the electronic device and the cables that connect the electronic device to the electrodes. (**b**) Internal side of the socks: equipped with five pockets for placing the TA, GL, and reference electrodes. (**c**) User wearing sock: correct placement of electrodes enables the measurement of the EMG signal from selected muscles.

To seamlessly integrate the electrodes into the sock, five small pockets made of elastic fabric were created and sewn together with the cables that connect the electrodes to the electronic device. These pockets were strategically positioned to ensure effective monitoring of the GL and TA muscles. A prototype of the sock is shown in Figure 2.

For signal processing of the EMG signals captured by the sensorized socks, an algorithmic framework was developed and tested on an embedded PC equipped with a Bluetooth connection.

### 2.2. Data Acquisition and Elaboration

**Figure 3:**
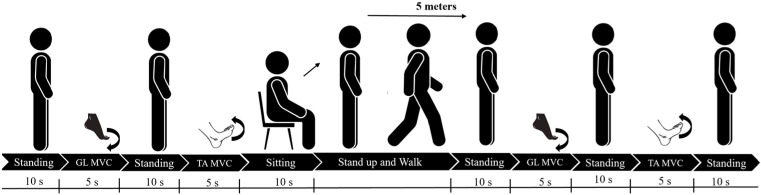
Acquisition protocol. Each volunteer performed the activities shown in the figure for the time indicated, where GL MVC and TA MVC are defined as the maximum voluntary contraction of the Gastrocnemius muscle and Tibialis muscle, respectively.

To evaluate the electromyography signals coming from the device, five young healthy subjects of different ages (28.7 ± 7.1 years), weights (67.3 ± 8.5 kg), heights (1.73 ± 0.3 m), and sex (3 males and 2 females) simulated different motor tasks such as standing, voluntary contraction of the TA and GL muscles, sitting, and walking, under controlled laboratory conditions. The maximum voluntary contraction (MVC) of the GL muscle was achieved by plantar flexion of the ankle, while the MVC of the TA was obtained by dorsiflexion of the ankle. All the participants provided written consent to participate in this study. The protocol is shown and described in Figure 3.

The participants in the experiment simulated the aforementioned events three times, by first using commercial pre-gelled electrodes, followed by the sock, ensuring the same monitoring points through markers placed on the target leg muscles. The areas on which the electrodes were applied were shaved and cleaned with isopropyl alcohol to minimize impedance. The data collected were then analyzed to assess the efficiency of the developed wearable system by comparing them with data from the pre-gelled electrodes. Data acquisition was performed using a gain setting of 12 and a sampling rate of 1000 Hz on the Shimmer sensing transmission module.

Data analysis was carried out using the MathWorks Matlab platform [39]. The key algorithmic steps for extracting the typical indices used to evaluate lower limb muscle contractions included (1) pre-processing, (2) calibration, and (3) feature extraction.

The pre-processing phase involved three main steps: (a) noise reduction, (b) EMG signal enveloping, and (c) data normalization. To minimize baseline noise and disturbances caused by electrode movement, a 4th order Butterworth band-pass filter (20 Hz to 450 Hz) was applied to the raw signals [40]. Next, the signal’s linear envelope was calculated through full-wave rectification and a low-pass Butterworth filter with a 10 Hz cut-off frequency.

### 2.3. Hybrid Polymer Electrode Preparation

**Figure 4:**
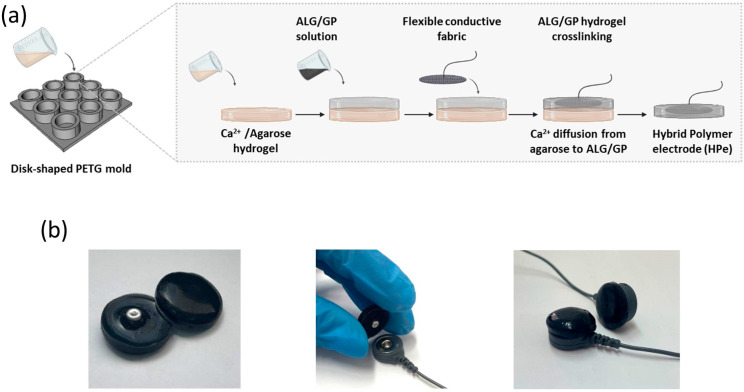
(**a**) Schematic representation of hybrid polymer electrode (HPe) production. (**b**) Photographs of fabricated HP electrodes and their connection to a commercial snap-on cable.

The production of alginate-based hydrogel electrodes follows a sequential step process, as schematically reported in Figure 4. First, alginic acid sodium salt (Sigma-Aldrich, St. Louis, MO, USA) was dissolved in distilled water under stirring for 2 h at 60 °C to obtain a 4 wt/vol (%) alginate solution (ALG). Next, 40 mg/mL graphite powder (GP) was dispersed in the alginate solution and constantly stirred for 3 h. A disk-shaped PETG mold (D = 17 mm, h = 10 mm) was designed using an open-source design software (FreeCAD 0.21) and then produced using the M300 plus 3D printer from Zortrax, Olsztyn, Poland. Subsequently, 1% agarose powder (Sigma-Aldrich) was dissolved in a 0.1M CaCl_2_ solution, boiled, and gelled in the PETG mold at room temperature. The ALG/GP solution was then poured on the top of the agarose disks, and a flexible conductive fabric (knitted superlight conductive fabric, InnTex, Texe srl, Italy) containing an Ag/AgCl snap button was incorporated into the hydrogel matrix. Alginate was chemically cross-linked at 4 °C overnight, through the radial diffusion of calcium ions from the agarose disk below. The electrodes obtained after gelling were 2.5 mm high, with a surface area of 5.1 cm^2^.

### 2.4. Electrode–Skin Impedance Characterization

The electrode–skin impedance measurements were performed using a three-electrode configuration by a potentiostat/galvanostat (Ivium Vertex.One, Ivium Technologies B.V., Eindhoven, The Netherland). The impedance characterizations were conducted with the produced hybrid polymer electrodes and then compared with commercial ECG hydrogel electrodes (CleartraceTM1700-O30 Diagnostic ECG electrodes, ConMed Corporation, Utica, NY, USA; geometric surface area = 5.1 cm^2^). Before the measurements, the selected area of the arm was prepared with alcohol, and the electrodes were attached. The impedance changes with respect to the potential were recorded at 20 Hz in the potential range from −0.1 to 0.2 V. Impedance spectra were recorded using a 10 mV sinusoidal input driving voltage at a frequency range from 0.1 Hz to 104 Hz. The impedance data were fitted with an optimized equivalent electric circuit using Zview 3.5h software (Scribner Associates Inc., Southern Pines, NC, USA).

## 3. Results

### 3.1. Electrode–Skin Impedance

The electrodes were fabricated in a circular shape, as schematically depicted in Figure 4. Alginate polymer with good biocompatibility [41] was used as the hydrogel matrix mixed with graphene and the flexible fabric to provide high conductivity and mechanical stability to the electrode. An agarose hydrogel prepared in a calcium solution was used as a sacrificial gel to allow a slow diffusion of the calcium ions toward the alginate solution and homogenous cross-linking of the ALG/GP hydrogel.

**Figure 5:**
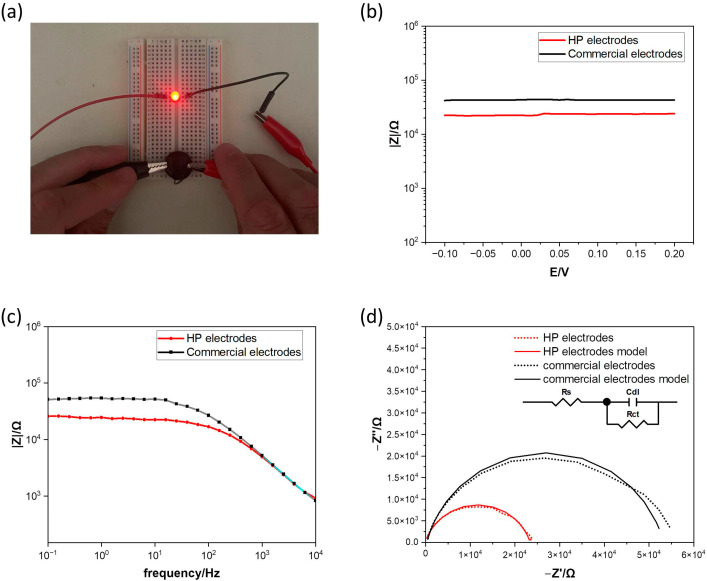
(**a**) LED test with HP hydrogel electrode; (**b**) impedance versus potential of HP hydrogel electrodes and commercial hydrogel electrodes; (**c**) impedance versus frequency of HP electrodes and commercial electrodes; (**d**) Nyquist plot and equivalent circuit model used for fitting. Dashed lines represent measured experimental data; solid lines represent the fitted curves.

The electrical conductivity of the hydrogels was measured with a multi-meter, and the average electrical resistance in DC was 0.98 ± 0.01 kΩ. The conductivity of the HP electrodes was also demonstrated in the LED test by connecting the hydrogel in series with an LED lamp and applying 3 V from a common power supply (Figure 5a). Another important parameter to characterize is the electrode–skin impedance, as it represents the impedance during EMG measurements. The electrode–skin impedance generally has a strong influence on the quality of bioelectrical signal acquisition, due to the attenuation effect from the stratum corneum and the epidermis [42]. To minimize noise sensitivity and guarantee high-quality recording, flexible and low-impedance electrodes are recommended. To assess the performance of HP electrodes for EMG sensing, electrode–skin contact impedance measurements were performed. Impedance data of the HP electrodes and the commercial hydrogel electrodes were recorded under the same experimental conditions on the arm of a volunteer. A first analysis was conducted to investigate the influence of the potential on the impedance. As shown in Figure 5b, the impedance values were not affected by the potential amplitude but a difference between the electrode types was visible. The HP electrodes presented a lower impedance amplitude than the commercial electrodes. The results of the impedance spectroscopy in the frequency range of 0.1–104 Hz are shown in Figure 5c,d. The impedances at 10 Hz of the HP electrodes and the commercial electrodes were 22.5 kΩ and 51.8 kΩ, respectively. The reduction in the impedance is visible across all frequencies and is due to the high conductance of the HP electrodes compared with that of the commercial electrodes. The Nyquist plots were fitted with a simple Randles cell, frequently used to model the impedance of the electrodes versus skin [43]. The equivalent electrical circuit is reported in the inset in Figure 5d and consists of R_s_ representing the ohmic resistance, R_ct_ the charge transfer resistance, and C_dl_ accounting for the capacitance of the double layer. Table 3 summarizes the values of the fitted equivalent circuit components. The plot in Figure 5d and the relatively small error of the fitting results suggest that the proposed equivalent circuit model adequately represents the electrode–skin impedance with a good agreement with the experimental data. The HP electrodes showed a lower impedance at the skin interface and are, therefore, suitable for EMG measurements.

### 3.2. Socks’ Performance

To compare the signals acquired with the sock and commercial pre-gelled electrodes and to assess the relationship between the two, the correlation coefficient R was calculated based on data collected using the protocol described in Section 2.2 and processed as outlined above. The correlation coefficient matrix for two random variables, A and B, represents the pairwise correlation between variables. Since A and B are always perfectly correlated with themselves (diagonal values equal 1), the correlation matrix R is as follows:

(1) $$R=\left(\begin{array}{cc} 1 & \rho (A,B)\\ \rho (B,A) & 1 \end{array}\right)$$

where ρ(*A*,*B*) = ρ(*B*,*A*) is the Pearson correlation coefficient [44].

**Figure 6:**
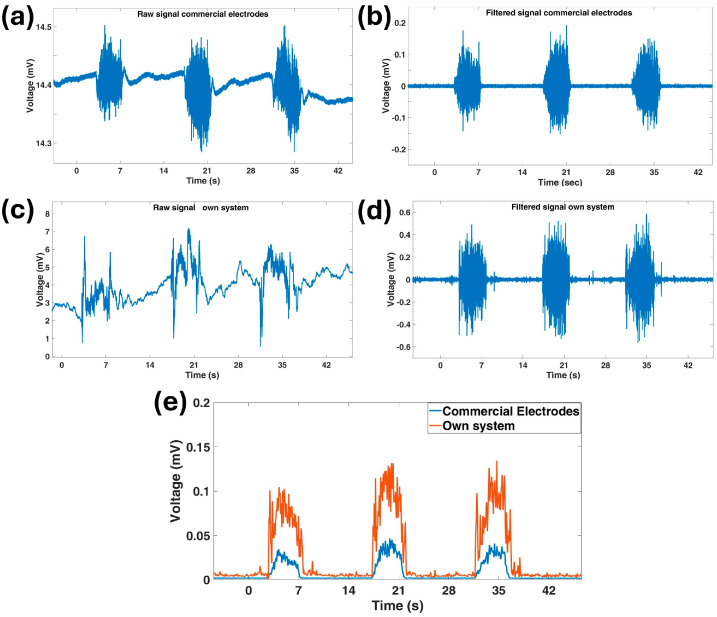
(**a**) Raw and (**b**) processed signals recorded with the commercial system; (**c**) raw and (**d**) processed signals captured using the in-house system; and (**e**) a comparison of the processed signal, measured using commercial electrodes and in-house-developed stockings during the execution of three repetitions of 5 s of MVC of the Tibialis Anterior muscle followed by 10 s of rest.

The correlation coefficient measures the linear dependence between two random variables. The average correlation coefficient R across all sessions with the five participants was 0.87, indicating a strong linear relationship between the two acquisition systems. This suggests that the signals obtained using the sock are highly comparable with those from the commercial system. The correlation between the EMG signals can also be visually assessed, as demonstrated in Figure 6, which presents examples of both raw and processed signals acquired using the commercial system and our own. The measurements were obtained by performing the following sequence of actions: about 5 s of maximum voluntary contraction (MVC) of the Tibialis Anterior muscle followed by 10 s of rest, repeated three times. The recordings were acquired while wearing both the smart sock and the pre-gelled electrodes placed adjacently, using the same acquisition system to ensure direct comparability. Moreover, the signals produced by our system exhibited a higher amplitude than that of the commercial system, albeit with increased noise.

To further assess signal quality, the signal-to-noise ratio (SNR) of the signals acquired during the muscle contraction tests was evaluated. The average SNR was approximately 24 dB, which is sufficient for reliable electromyographic signal analysis [45].

**Figure 7:**
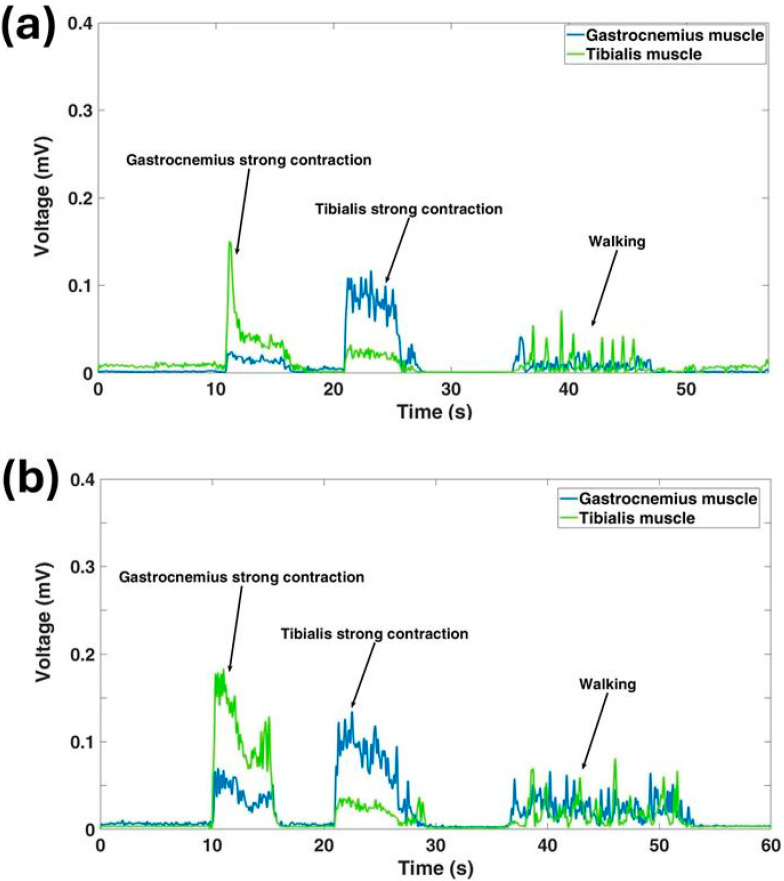
Example of signal patterns captured from the Gastrocnemius and Tibialis muscles during walking and strong muscle contraction using (**a**) the commercial electrodes and (**b**) the sock system.

Figure 7 presents the signal patterns recorded from the Gastrocnemius and Tibialis muscles during walking and strong muscle contractions, using the sock system and commercial electrodes, in two different users. A visual comparison reveals that these signals closely resemble those typically obtained from standard commercial systems for the same activities.

## 4. Discussion and Conclusions

This paper presents a pioneering approach in the development of a smart surface electromyography-based wearable device, specifically in the form of smart socks, designed to monitor muscle activation in the Gastrocnemius Lateralis and Tibialis Anterior muscles. The primary objective of this study is to enhance the efficiency and usability of surface electromyography for monitoring lower limb conditions to enable the early detection of abnormalities such as sarcopenia, fall risk, and postural issues.

One of the standout features of this research is the use of hybrid polymer electrodes, which address many of the limitations posed by traditional Ag/AgCl gel electrodes. This study showed that these biocompatible hydrogel electrodes, integrated into the fabric of the smart socks, exhibited a lower impedance at the interface with the skin. This is particularly beneficial when capturing dynamic muscle activity during walking or other movements, making the system suitable for real-life fall risk evaluations and muscle monitoring.

This research also successfully showcased the effectiveness of the smart socks in providing reliable data. Comparisons between the signals obtained from these hydrogel-based electrodes and standard pre-gelled electrodes revealed a high correlation, with an average Pearson correlation coefficient of 0.87. This indicates that the smart socks can serve as a viable alternative to traditional methods without sacrificing signal quality. Furthermore, the device demonstrated sufficient signal-to-noise ratio values, confirming the system’s capability to capture high-quality EMG signals.

Another important contribution of this work is the integration of all electronic components within the sock, which enables simple, wireless data acquisition and transmission.

However, despite these promising outcomes, this study also recognizes the challenges that need to be addressed to optimize the smart socks for wider clinical and everyday use. Chief among these is the need for further refinement of the hydrogel’s durability, as prolonged use could lead to a gradual degradation in the adhesive and conductive properties of the electrodes. This would require periodic replacement, which might limit long-term applicability. Moreover, the complexity of manufacturing the hydrogel electrodes could potentially drive-up production costs, although research suggests that scaling up the production process could mitigate these concerns.

Looking forward, the next steps of this research should focus on testing smart socks in different populations, particularly in the elderly who are at higher risk for falls and related conditions. In addition, a miniaturized ad hoc data acquisition hardware system will be developed that is less invasive and more energy efficient to improve the energy efficiency of the system, as extending the battery life and the operational range will be critical for continuous, long-term use. Enhancing these features will ultimately improve the practicality of the smart socks for real-world applications, enabling users to monitor muscle activity and health indicators consistently over time. Once the system has been further optimized, dedicated data collection campaigns can be conducted to develop software frameworks capable of detecting more complex conditions, such as the recognition of postural anomalies, fall risk, and sarcopenia.

The use of a compression sock helps minimize sensor displacement, providing better electrode stability during movement. In addition, appropriate machine learning techniques for muscle state assessment or abnormality detection can improve the robustness of the system against small sensor displacements. These aspects will be the subject of future developments through which the dataset will be expanded with more complex movements, and the signal analysis software framework will be further developed.

In conclusion, this research demonstrates that the integration of conductive hybrid polymer electrodes into smart wearable systems like socks has great potential to revolutionize how we monitor and assess muscle function, recording high-quality EMG signals. The smart socks developed in this study offer a user-friendly, cost-effective solution for continuous electromyography monitoring, making them particularly well suited for fall risk evaluation and muscle rehabilitation. With further optimization and testing, this technology could become a staple in healthcare, improving quality of life for the elderly and individuals with neuromuscular conditions.

## Figures and Tables

**Table 2:** Technical specifications of the Shimmer3 EMG device.

Features	
Data rate	Software configurable (125, 250, 500, 1000, 2000, 4000, 8000 SPS)
Input differential dynamic range	Approx. 800 mV (for gain = 6)
Bandwidth	8.4 kHz
Ground	Wilson-type driven ground
Input protections	ESD and RF/EMI filtering; current limiting; inputs include defibrillation protection
Weight	31 g
Dimensions	65 × 32 × 12 mm
EEPROM memory	2048 bytes
Processing	MSP 430 microcontroller (24 MHz, 16 Bit)
Communication	Bluetooth—RN4678
Storage	Integrated 8 GB microSD card slot
Battery	450 mAh rechargeable Li-ion
Integrated 3-axis accel,	ICM-20948 (Accel. range ±2 g, ±4 g, ±8 g, ±16 g)

**Table 3:** Average fitted parameter values of HP and commercial electrodes.

	R_s_	C_dl_	R_ct_
HP electrodes	0.375 ± 0.01 kΩ	27.78 ± 0.1 nF	22.43 ± 0.6 kΩ
Commercial electrodes	0.588 ± 0.012 kΩ	32.57 ± 1.1 nF	52.25 ± 3.1 kΩ

## Data Availability

The data presented in this study are available on request from the corresponding author. The data are not publicly available due to restrictions (they contain information that could compromise the privacy of research participants).
